# Interactions Between Genes From Aging Pathways May Influence Human Lifespan and Improve Animal to Human Translation

**DOI:** 10.3389/fcell.2021.692020

**Published:** 2021-08-19

**Authors:** Svetlana Ukraintseva, Matt Duan, Konstantin Arbeev, Deqing Wu, Olivia Bagley, Arseniy P. Yashkin, Galina Gorbunova, Igor Akushevich, Alexander Kulminski, Anatoliy Yashin

**Affiliations:** Biodemography of Aging Research Unit, Duke University, Durham, NC, United States

**Keywords:** aging pathways, animal to human translation, heterogeneity of longevity, genetic interactions, statistical epistasis, stress response, human lifespan, aging genes

## Abstract

A major goal of aging research is identifying genetic targets that could be used to slow or reverse aging – changes in the body and extend limits of human lifespan. However, majority of genes that showed the anti-aging and pro-survival effects in animal models were not replicated in humans, with few exceptions. Potential reasons for this lack of translation include a highly conditional character of genetic influence on lifespan, and its heterogeneity, meaning that better survival may be result of not only activity of individual genes, but also gene–environment and gene–gene interactions, among other factors. In this paper, we explored associations of genetic interactions with human lifespan. We selected candidate genes from well-known aging pathways (IGF1/FOXO growth signaling, P53/P16 apoptosis/senescence, and mTOR/SK6 autophagy and survival) that jointly decide on outcomes of cell responses to stress and damage, and so could be prone to interactions. We estimated associations of pairwise statistical epistasis between SNPs in these genes with survival to age 85+ in the Atherosclerosis Risk in Communities study, and found significant (FDR < 0.05) effects of interactions between SNPs in *IGF1R*, *TGFBR2*, and *BCL2* on survival 85+. We validated these findings in the Cardiovascular Health Study sample, with *P* < 0.05, using survival to age 85+, and to the 90th percentile, as outcomes. Our results show that interactions between SNPs in genes from the aging pathways influence survival more significantly than individual SNPs in the same genes, which may contribute to heterogeneity of lifespan, and to lack of animal to human translation in aging research.

## Introduction

Many genes and their products have individually been found to significantly influence aging and survival traits in experimental models, including yeast, nematodes, flies, and mice. Genes for growth hormone and IGF1 receptors, FOXO transcription factors, target of rapamycin, p16, klotho, sirtuins, and some others, were repeatedly featured in experimental studies of aging and lifespan extension [e.g., Johnson et al., 2002, 2013; Braeckman and Vanfleteren, 2007; Kenyon, 2010; Pavlatou et al., 2016; Uno and Nishida, 2016; Bartke and Quainoo, 2018; Singh et al., 2019; Tian et al., 2019; also reviewed in Ukraintseva et al. (2021)]. However, the majority of such genes have not been consistently replicated in humans, with few exceptions such as, e.g., *FOXO3* and *KL* (Arking et al., 2005; Willcox et al., 2008; Zeng et al., 2010; Nygaard et al., 2013, 2014; Soerensen et al., 2016; Donlon et al., 2017; Revelas et al., 2018; Morris et al., 2019).

**FIGURE 1:**
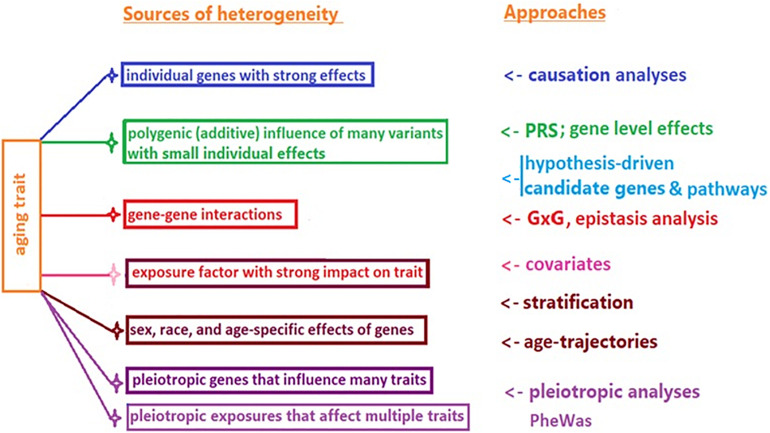
Common sources of heterogeneity of aging and longevity related traits, and approaches to their study.

The potential reasons for this lack of animal to human translation may include a highly conditional character of genetic influence on lifespan, and heterogeneity of longevity. The former refers to the possibility of different (or even antagonistic) influence of the same genetic variant on survival in different species/strains, or in the same species in different age groups and environments (de Magalhães, 2014; Ukraintseva et al., 2016). The latter (heterogeneity) implies that the high chances of survival to extreme age could be achieved through different genetic pathways, individual genes, additive polygenic effects, genetic interactions (G × G), and gene–environment interactions (G × E), among other factors (Figure 1).

A careful look at genes (and their products) that have been consistently featured in experimental aging research reveals that the majority of such genes belong to just a few well-known “aging pathways” that regulate outcomes of the cell responses to stress and damage, such as (using human orthologs names):

• IGF1/AKT/FOXO3 – mediated cell survival, growth, and DNA repair;
• TP53/P21/P16 – mediated apoptosis, growth arrest, senescence, and autophagy;
• mTOR/S6K – mediated autophagy, cell survival, and growth.

**FIGURE 2:**
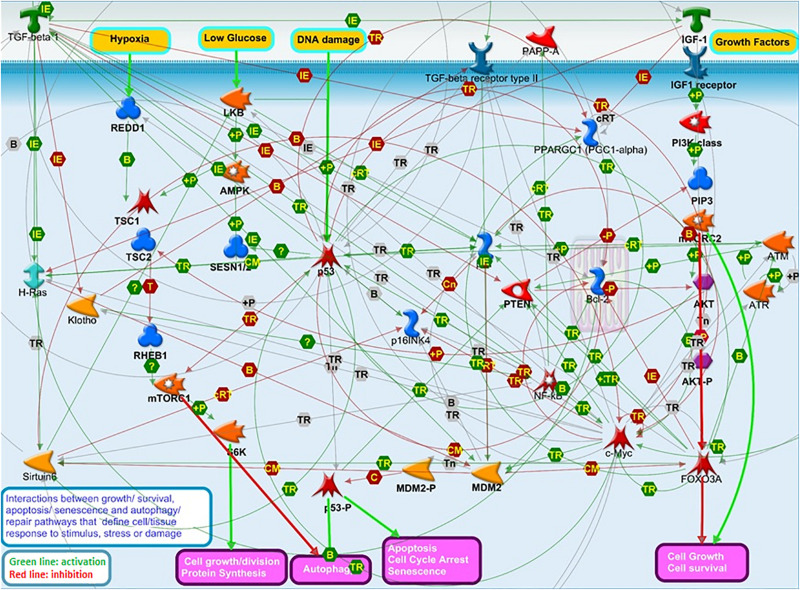
The interplay among “aging” pathways that have been a major focus of aging research over the last decades: IGF1/AKT/FOXO3 growth signaling, TP53/P21/P16 apoptosis and senescence, and mTOR/S6K autophagy and survival pathways. This Figure illustrates the complexity of the interactions between these pathways regulating cell responses to stress and damage, which may in turn impact tissue resilience, and, ultimately, influence organismal survival and lifespan. This Figure was prepared using MetaCore Pathway Map Creator (PMC) tool (Dubovenko et al., 2017) from *Clarivate Analytics*. The PMC allows to connect gene products selected by the user in a picture showing the molecular interactions occurring between members of respective cellular processes and pathways. The latter are defined, annotated, and manually curated by *Clarivate Analytics* scientists based on the up-to-date literature and pathway libraries. Main protein classes shown on Figure 2 are annotated in Supplementary Table 3.

These pathways closely biologically interact and work in concert to decide on outcomes of cell responses to stress and damage, such as apoptosis, senescence, growth, division, autophagy, and repair, which may impact tissue resilience and, in turn, organismal survival and longevity (Levine et al., 2006; Feng et al., 2007; Tran et al., 2014; Bitto et al., 2015; Werner et al., 2016; Ukraintseva et al., 2021; Figure 2). We, therefore, propose that the interplay between genes in these pathways may influence human lifespan.

In this paper, we selected candidate genes that belong to these aging-related pathways *and* have also been featured in experimental aging research, and investigated the effects of interactions (statistical epistasis) between SNPs in these genes on survival in human data.

## Materials and Methods

### Data

We used existing human data collected in the Atherosclerosis Risk in Communities (ARIC) and the Cardiovascular Health Study (CHS). The ARIC cohort of 15,792 participants, aged 45–64 at baseline received an extensive examination at 5 clinic visits conducted between 1987 and 2013, and yearly follow-ups assessing health status by telephone. By the time of last exam, almost all participants were older than 65. The CHS original cohort of 5,201 participants aged 65+ at baseline in 1989–1990 has been followed with annual clinic examinations till 1999, and then for another 20 years (2000-present) with twice yearly telephone contacts and additional examination in 2005–2006. Genetic, phenotypic, and survival information collected in these studies has been provided to us through the NIH supported portal dbGaP upon request for controlled access. We used version 5 of the ARIC data (dbGaP accession phs000280), and version 7 of the CHS data (dbGaP accession phs000287), including the genotyping data from the Candidate Gene Association Resource (CARe). The CARe covers ∼49K SNPs in ∼2,100 candidate health-related genes (with increased coverage in CVD-related loci) genotyped on the Illumina ITMAT-Broad-CARe (IBC) chip designed to be inclusive of the intronic, exonic, untranslated regions (UTRs) and ∼5 kb of the promoter regions (Keating et al., 2008; Musunuru et al., 2010). The IBC chip utilizes a tagging approach to capture the genetic diversity across these candidate genes, informed by GWAS, expression quantitative trait loci, pathway-based approaches and comprehensive literature searching, prioritized based on consensus by the investigators, as described in detail in Keating et al. (2008). Using the same array (IBC chip) in different CARe studies, including ARIC and CHS, aims to facilitate harmonization and replication of gene–phenotype associations across the studies (Musunuru et al., 2010). We used ARIC as the discovery dataset, and CHS as the validation set because the total number of SNPs available in the candidate genes of interest was lower in ARIC than in CHS after quality control (QC) (863 vs. 1,053), and the ARIC sample also included more Black participants (Table 1A).

**TABLE 1:** Study sample, candidate genes, and outcome phenotypes.

**Dataset**	**Males**	**Females**	**Black (%)**	**White (%)**	**SNPs (within candidate genes ±1 kb)**
**A. Study sample by sex, race, and number of SNPs, after QC**					
CHS	2,201	2,955	15.2	84.1	1,053
ARIC	5,962	7,350	26	74	863 (overlapped with SNPs in ARIC)
**B. Selected candidate genes from the aging-related pathways that have been featured in experimental research (human ortholog names, by the HUGO Gene Nomenclature Committee)**					
IGF1/AKT/FOXO3 growth signaling:					
*AKT1, ATM, FOXO1, FOXO3, GHR, HIF1A, IGF1, IGF1R, PIK3CA, PIK3CB*;					
TP53/P21/P16 apoptosis/senescence:					
*BAX, BCL2, CDK4, CDK6, CDKN1A (P21)*, *CDKN2A (P16)*, *CDKN2B (P15), FAS, TP53*;					
mTOR/S6K mediated autophagy/survival:					
*AMPK subunits (PRKAA1, PRKAA2, PRKAB2, PRKAG1, PRKAG2), SIRT1, RPS6KB1 (S6K), TSC2*;					
Genes broadly involved in the cross-talk between the aging pathways:					
*KL, MYC, NFKB1, NFKB2, PPARGC1A, PTEN, TGFB1, TGFBR2.*					
**C. Outcome phenotypes**					
*Survival 85*+ (in ARIC and CHS):					
Survived to age 85+ (1) vs. died before age 85 (0)					
*Survival to the 90th percentile* (in CHS only, see section “Phenotypes” for detail):					
Survived to *age corresponding to 10% of the longest lived (1) vs. died before that age (0)					

*The ages corresponding to 10% of the longest lived, based on sex- and race-specific life tables for the US population: Black and White women – 95 years; Black men – 91 years; White men – 93 years.

### Candidate Genes

To explore the possibility that genes from the major aging pathways (IGF1/AKT/FOXO3, TP53/P21/P16, and mTOR/S6K mediated) may influence human lifespan as result of their interplay rather than independently, we selected the set of candidate genes (Table 1B) that belong to these pathways *and* have also been featured in aging research, as genes or their products (Braeckman and Vanfleteren, 2007; Feng et al., 2007; Tsai et al., 2008; Ghosh et al., 2010; Kenyon, 2010; Johnson et al., 2013; Nojima et al., 2013; Ortega-Molina and Serrano, 2013; Tran et al., 2014; Cetrullo et al., 2015; Pavlatou et al., 2016; Uno and Nishida, 2016; Yuan et al., 2016; Donlon et al., 2017; Bartke and Quainoo, 2018; Morris et al., 2019; Singh et al., 2019; Blasiak et al., 2020; Zhang et al., 2020; Tabibzadeh, 2021). Majority of these genes are involved in cell/tissue responses to stress and damage that can contribute to the body’s ability to recover (resilience) and through this to its ability to survive to the oldest old age (Ukraintseva et al., 2021). For the epistasis analysis, we selected 863 SNPs located in these genes based on the list of the SNPs genotyped on the IBC chip and available in both ARIC and CHS CARe data after QC (Table 1A and Supplementary Table 2).

### Phenotypes

As the main outcome phenotype, we used a binary survival trait: *survived to age 85*+ *(1) vs. died before age 85 (0)*. The choice of this phenotype was motivated by our working hypothesis that the age around 85 may be a turning point in the course of aging, characterized by trade-off-like changes in the effects of certain risk factors on survival (Ukraintseva et al., 2016). Our earlier studies suggested that risks of many major diseases start to decline or level-off (after prior increase) around that age (e.g., Ukraintseva and Yashin, 2003; Akushevich et al., 2012), which could be due to selection, under-diagnosis, or the aging itself changing the effects of respective risk factors antagonistically, so that they may negatively affect health and survival chances before the age 85 but become somewhat protective afterwards (Ukraintseva et al., 2016). So, identifying the genetic factors that can influence survival at ages 85+ vs. 85− was of particular interest to us.

We also included an additional phenotype of survival in the CHS data analysis, using the age cut-off based on survival to the 90th percentile (corresponding to 10% of the longest lived in the US population). Our discovery dataset (ARIC) is not suitable for investigating such outcome because it includes only a few people older than 90. However, the validation dataset (CHS) contains a considerably larger sample of individuals aged 90+ (600), allowing to include the “90th percentile” survival outcome in the analysis. We used population life tables from the US Centers for Disease Control and Prevention^1^ to determine the ages corresponding to the survival to the 90th percentile, by sex and race: 95 years for Black and White females, 91 years for Black males, and 93 years for White males. These ages were used to construct respective phenotypes of survival (Table 1C) and include them in the validation analysis in the CHS, using genes that were discovered in ARIC (see section “Results”).

### Statistical Analysis

The QC procedures were based on Anderson et al. (2010) and Marees et al. (2018), and were performed before the epistasis analysis. We removed duplicates, people who failed in sex check, individuals with >5% missing SNPs, and SNPs with the genotyping rate lower than 95% and minor allele frequency (MAF) lower than 1%. In addition, SNPs that failed the Hardy–Weinberg test (*P*-value < 10^–10^) were also excluded. Study sample and numbers of individuals and SNPs available for the analysis are shown in Table 1A and in Supplementary Tables 1.1, 1.2.

Using all genotyped SNPs available after QC in the selected candidate genes (Supplementary Table 2), we estimated associations of a pairwise SNP × SNP epistasis with survival 85+ in ARIC data (discovery set) and validated the findings in CHS data, using the binary phenotypes of survival to the age 85+ and to the 90th percentile as outcomes (Table 1C; see section “Phenotypes” for detail). For this analysis, we used INTERSNP software for statistical epistasis (Herold et al., 2009), and logistic regression model with covariates, including: birth cohort *z*-score, education level (0 – below high school, 1 – high school, 2 – above high school), and smoking status (1 – ever smoked, 0 – never smoked). We also included the first two principal components to control for possible population stratification. All analyses were stratified by sex (males and females) and race (Blacks and Whites). Also, to address a common concern in a high dimensional study that small sample sizes in some groups may lead to zero data points in contingency table cells and to increase in type I errors, we applied an additional selection criterion in INTERSNP, requiring the sum of the four genotype combinations containing minor alleles (MA) (2MA–2MA, 2MA–1MA, 1MA–2MA, 1MA–1MA) to be at least 20, to ensure that there are sufficient numbers of individuals in the analysis in each race and sex group. We also estimated and compared the effects of individual SNPs in the selected candidate genes with the effects of the SNP × SNP interactions on the same survival outcomes.

## Results

### Survival to Age 85+

We first estimated associations of the interactions between SNPs in the candidate genes shown in Table 1B with *survival to age 85*+ in the discovery set (ARIC), and selected results corrected for multiple comparisons (FDR < 0.05) to reduce chances of false positive findings. Of those, we selected the SNP pairs that also influenced *survival to age 85*+ in the validation set (CHS) with at least conventional significance (*P*-value < 0.05). These results are shown in Table 2. One can see that the interaction between rs939626 (*IGF1R*) and rs3773663 (*TGFBR2*) is significantly (*P*-value = 2.1E−06; FDR < 0.05) associated with *survival to age 85*+ in White women in ARIC, which is confirmed in CHS with conventional significance (*P*-value = 0.03) and the same direction of the effect. The associations of individual SNPs in these same genes with *survival to age 85*+ didn’t reach a significance at the 0.05 level in this data (Table 2, *P*-v1 and *P*-v2 columns).

**TABLE 2:** Results of associations of interactions between SNPs in selected candidate genes from aging pathways (Table 1B) with survival to age 85+ in ARIC and CHS CARe.

**Data**	**Ch1**	**SNP1**	**MAF1**	***P*-v1**	**Gene1**	**Ch2**	**SNP2**	**MAF2**	***P*-v2**	**Gene2**	**Race**	**S**	**OR**	***P*-value**	***N***
ARIC	3	rs3773663	A(0.42)	0.14	TGFBR2	15	rs939626	G(0.47)	0.07	IGF1R	W	F	0.26	*2.1E−06	1,430
CHS	3	rs3773663	A(0.41)	0.09	TGFBR2	15	rs939626	G(0.45)	0.12	IGF1R	W	F	0.75	0.03	1,519
ARIC	15	rs11247378	T(0.13)	0.96	IGF1R	18	rs956572	A(0.25)	0.73	BCL2	B	F	0.10	*3.9E−07	810
ARIC	15	rs11247380	A(0.28)	0.37	IGF1R	18	rs956572	A(0.25)	0.73	BCL2	B	F	0.35	5.5E−04	811
CHS	15	rs11247380	A(0.27)	0.32	IGF1R	18	rs956572	A(0.25)	0.20	BCL2	B	F	0.33	0.007	276

*Ch1 and Ch2, chromosome numbers for SNP1 and SNP2, respectively; SNP1 and SNP2, dbSNP Reference SNP numbers. All SNPs shown in this table are located in introns of corresponding genes. Note that the SNPs rs11247378 and rs11247380 are in LD in African Americans (D′ = 0.69; *R*^2^ = 0.13), according to LDlink (https://ldlink.nci.nih.gov/?tab=ldpair); MAF1 and MAF2, minor allele (and its frequency) for SNP1 and SNP2, respectively; *P*-v1 and *P*-v2, *P*-values for associations of individual SNPs with survival 85+, for SNP1 and SNP2, respectively; Gene1 and Gene2, gene names by the HUGO Gene Nomenclature Committee; Race: B, Black; W, White; S, sex; F, female; OR, odds ratio; *P*-value, unadjusted *P*-value for the association of SNP1 × SNP2 interaction with survival 85+. Asterisk before the *P*-value indicates results with FDR < 0.05 in discovery set (ARIC). N, number of people included in the analysis.*

Our analysis suggests that the double-carriers of major alleles of the two SNPs (i.e., individuals carrying both rs939626 AA *and* rs3773663 GG variants) have a higher probability to live to age 85+, compared to the carriers of other allelic combinations of these SNPs. Additional exploration using the Genotype-Tissue Expression (GTEx) eQTL Calculator^2^ revealed that, for each of these SNPs, being homozygous for major allele may result in a slightly (though not significantly) lower average expression of respective gene (IGF1R or TGFBR2). Although this tendency does not allow us to make a reliable interpretation, it, potentially, may point to benefits of downregulation of respective pathways for achieving the longer life. To further explore this, more data about the effects of these SNPs on gene expression is needed, which could become available in the future. We do not describe here the effects of the epistatic *interaction* between rs939626 and rs3773663 on gene/protein expression, since this information is not yet available in relevant bases. It is important to note, however, that the detection of statistical interaction between the two loci does not necessarily mean the existence of direct biochemical interaction between respective genetic products (see also the section “Discussion”).

A significant (*P*-value = 3.9E−07; FDR < 0.05) association with *survival to age 85*+ was found for the interaction between rs956572 (*BCL2*) and rs11247378 (*IGF1R*) in Black women in ARIC. This was supported by the result of interaction analysis between rs956572 and rs11247380 in Black women in both ARIC and CHS, with conventional *P*-values (Table 2). However, rs11247380 and rs11247378 (*IGF1R*) are in only moderate LD (D′ = 0.69; *R*^2^ = 0.13), therefore, the effect of the interaction between rs956572 and rs11247378 or rs11247380 on *survival 85*+ should be viewed as suggestive and has to be confirmed in further research.

The results in Table 2 also point to a potentially major role of the IGF1R gene in the non-additive multigenic regulation of lifespan in both Blacks and Whites. However, significant (FDR < 0.05) associations with *survival 85*+ were observed for the interactions that included different SNPs in IGF1R gene in Blacks and Whites. Such differences may be caused by, e.g., differences between Blacks and Whites in MAF and LD structures, or in histories of exposures to environmental and living conditions, among other factors.

### Survival to the 90th Percentile

Next, we selected all 355 SNPs available in data for the top interacting genes that influenced *survival 85*+ in the first step (i.e., TGFBR2, IGF1R, and BCL2, from Table 2), and estimated associations of the interactions between SNPs in these genes with *survival to the 90th percentile* (see Table 1C and section “Phenotypes”) in the CHS validation set. The strongest effect on *survival to the 90th percentile* in the CHS was observed for the interaction between rs4955189 (TGFBR2) and rs8034284 (IGF1R) in White males (*P*-value = 4.9E−06; FDR = 0.029). According to LDlink,^3^ rs4955189 is in moderate LD (D′ = 0.51; *R*^2^ = 0.22; Whites-CEU) with rs3773663 (TGFBR2) from Table 2, which means that the top G × G effects on *survival 85*+ and *survival to the 90th percentile* include correlated SNPs.

Notably, the *P*-value for the interaction between rs4955189 (TGFBR2) and rs8034284 (IGF1R) in the total sample (males and females combined) was less significant (7.5E−05) than that for White males (4.9E−06) despite the fact that the total sample was larger than that of the White males (2,235 vs. 1,141). This indicates that using a more restrictive model with sex as a covariate may not always benefit the analysis, if the associations differ by sex. Some experimental studies demonstrated sexually dimorphic effects of IGF1R (e.g., Corrochano et al., 2014; Xu et al., 2014), which may provide an additional support to the sex-stratified analysis of this gene.

We also found that the interaction between rs4955189 (TGFBR2) and rs7167580 (IGF1R) influenced *survival to the 90th percentile* in White males, as well as in the total CHS sample, with conventional significance (*P*-values = 0.009 and 0.003, respectively). This result may additionally support the interaction between rs3773663 (TGFBR2) and rs939626 (IGF1R) found in the discovery stage (Table 2), because rs7167580 is in moderate LD (D′ = 1; *R*^2^ = 0.35; Whites-CEU) with rs939626 (IGF1R).

## Discussion

Despite decades of aging research, the role of genetic interactions (G × G) in heterogeneity of human lifespan, and in animal to human translation, remains not fully understood. Several studies supported the involvement of G × G in human longevity (e.g., Zeng et al., 2010; Deelen et al., 2013; Fuku et al., 2017; Dato et al., 2018). Some focused specifically on the interactions between FOXO3 and other genes (Zeng et al., 2010; Fuku et al., 2017) and on relevant biology, such as DNA damage response (Tsai et al., 2008). Other researchers (Deelen et al., 2013) applied the pathway-based geneset approach to evaluating the joint effect of SNPs in genes from aging pathways on longevity in humans, and yielded results suggesting a major impact of the genetic variation in the IGF1 signaling pathway.

Dato et al. (2018) investigated the synergic SNP × SNP interactions in nonagenarians compared with controls aged 46–55 years, using tagging SNPs in 140 genes belonging to three candidate pathways (insulin/insulin-like growth signaling, DNA repair, and pro/antioxidant ones). They found the most significant interactions (FDR < 0.0001) between rs12437963 (IGF1R) and rs6067484 (PTPN1), as well as between rs2078486 (TP53) and two other genes (Dato et al., 2018). Results of our study seem to be in line with their findings, despite the difference in approach to the epistasis analysis between the two studies [INTERSNP in our case, and the multidimensional reduction (MDR) approach in Dato et al. (2018)]. In our study, we found a significant (FDR < 0.05) interaction between rs939626 and rs3773663 (Table 2), and it turns out that rs939626 is in LD (albeit a modest one: D′ = 0.79; *R*^2^ = 0.10) with rs12437963, the SNP in IGF1R gene that was involved in the top significant SNP × SNP interaction in the Dato et al. (2018). The fact that the top SNPs found in the two studies are correlated may additionally support the results of both these studies.

A central role of the communication between the P53–IGF1–AKT–mTOR pathways in regulating the cell growth, proliferation, and death, was suggested by Levine et al. (2006) about 15 years ago. Specifically, the authors pointed to a major significance of the interplay between cell survival and apoptosis in human lifespan. Results of our study support this view. They suggest that the interactions between IGF1R and TGFBR2, as well as BCL2, may influence human lifespan. IGF1R is growth factor receptor, which can promote cell survival acting as anti-apoptotic agent or inductor of proliferation. TGFBR2 is also a growth factor receptor, which may show growth inhibitory effect, depending on context. BCL2 gene codes for a mitochondrial anti-apoptotic protein, which can promote cell survival, including that of cancer cells, by inhibiting the apoptosis induced by oxidative stress. Current evidence suggests that these genes, and respective proteins, can be concurrently up/down-regulated to influence cell survival, apoptosis, and proliferation (Mayeenuddin et al., 2010; Basu et al., 2012; Valenciano et al., 2012; Alsina-Sanchis et al., 2016), which might contribute to their propensity to the epistatic interactions.

It is important to stress, however, that detection of the association of genetic interactions with complex traits using statistical methods (a.k.a. statistical epistasis) does not necessarily mean that products of respective genes interact directly biologically. Statistical analysis can capture genetic interactions mediated by molecular products of many other genes. The data from experimental studies may bring additional light on possible players in such mediation. Evaluating their role in human survival could be a next step in the clarification of multigenic mechanism of lifespan regulation in humans.

It is also worth mentioning that the SNP rs956572 (BCL2) that interacted with SNPs in IGF1R gene in our analysis (Table 2), on itself has been intensively studied for more than a decade, and appears to be broadly involved in aging and AD-related traits (e.g., Salvadore et al., 2009; Uemura et al., 2011; Liu et al., 2013; Chang et al., 2018), which might contribute to its propensity to genetic interactions that influence human lifespan. One should note that individual associations of this SNP with survival did not reach statistical significance in our analysis (*P*-v2 in Table 2).

In summary, the results of this study suggest that interactions between genes from the aging-related pathways may influence survival in humans more significantly than individual polymorphisms in the same genes. The fact that IGF1R, TGFBR2, and BCL2 genes appear among the top results of the interaction analysis may point to a major role of the interplay between cell survival and apoptosis in determining human lifespan. The G × G interactions may contribute to the lack of animal to human translation in genetics of aging because the landscape of such interactions, and their role in the structure of heterogeneity of lifespan may differ across species and strains.

## Data Availability Statement

The data analyzed in this study is subject to the following licenses/restrictions: The data were made available for the secondary analyses relevant to this article *via* controlled access provided by dbGaP, NIH supported resource. We do not own this data. Requests to access these datasets should be directed to: https://www.ncbi.nlm.nih.gov/gap/.

## Ethics Statement

The studies involving human participants were reviewed and approved by Duke University IRB. Note that in this article, we analyzed only already existing genetic and phenotypic information previously collected in CHS and ARIC. These data were de-identified by providers before release based on their expert opinion, which is in line with Expert Determination method of the data de-identification recommended by the Health Insurance Portability and Accountability Act (HIPAA) Privacy Rule (https://www.hhs.gov/hipaa/for-professionals/privacy/special-topics/de-identification/index.html#standard). The data were made available for the secondary analyses relevant to this article through dbGaP, NIH supported online data base. dbGaP, however, requires local IRB approval in order to grant access to the data. Duke University IRB approval was, therefore, obtained before the start of the analyses. Written informed consent for participation was not required for this study in accordance with the national legislation and the institutional requirements.

## Author Contributions

SU conceived and designed the study, wrote the manuscript, and provided interpretation of the results, with input from other co-authors. MD prepared the data for analysis, with input from DW, OB, and GG. MD analyzed the data, with input from DW, OB, and KA. MD and KA wrote sections of the manuscript. DW, KA, AY, IA, APY, and AK contributed to selection and discussion of methods used in the manuscript. KA, MD, and AY contributed to interpretation of the results. All authors contributed to the article and approved the submitted version.

## Author Disclaimer

The content is solely the responsibility of the authors and does not necessarily represent the official views of the National Institutes of Health.

## Conflict of Interest

The authors declare that the research was conducted in the absence of any commercial or financial relationships that could be construed as a potential conflict of interest.

## Publisher’s Note

All claims expressed in this article are solely those of the authors and do not necessarily represent those of their affiliated organizations, or those of the publisher, the editors and the reviewers. Any product that may be evaluated in this article, or claim that may be made by its manufacturer, is not guaranteed or endorsed by the publisher.

## Footnotes

1
https://www.cdc.gov/nchs/data/nvsr/nvsr68/nvsr68_04-508.pdf

2
https://gtexportal.org/home/testyourown

3
https://ldlink.nci.nih.gov/?tab=ldpair

## Supplementary Material

Supplementary Table 1.1 Total numbers of individuals and SNPs available in data, before and after QC.

Supplementary Table 1.2 Numbers of individuals used in the analyses, by age, sex, race, covariates, and comorbidity status.

Supplementary Table 2 SNPs in the candidate genes available for this study after QC.

Supplementary Table 3 Main protein/metabolite classes shown in Figure 2 in the text.
