# Supplementary material for: Interactions Between Genes From Aging Pathways May Influence Human Lifespan and Improve Animal to Human Translation
Source: Front Cell Dev Biol. 2021 Aug 19;9:692020. doi: 10.3389/fcell.2021.692020 (PMC8417405; doi:10.3389/fcell.2021.692020)
Supplement: Supplementary Table 1.1 — Total numbers of individuals and SNPs available in data, before and after QC. [file Data_Sheet_1.docx]

**SUPPLEMENTARY MATERIALS**

**Supplementary Table 1.1 |** Total numbers of individuals and SNPs available in data, before and after QC

| **Dataset** | **Males**  (after QC/before QC) | **Females**  (after QC/before QC) | **Males+Females**  (after QC/before QC) | **all SNPs on IBC Chip**  (after QC/before QC) |
| --- | --- | --- | --- | --- |
| CHS | 2,201/2,255 | 2,955/3,024 | 5,156/5,279 | 34,387/49,094 |
| ARIC | 5,962/6,279 | 7,350/7,889 | 13,312/14,248 | 28,800/47,359 |

**Supplementary Table 1.2 |** Numbers of individuals used in the analyses, by age, sex, race, covariates, and comorbidity status

| **Data**  (by sex and race) | **Survival** | **N** | **Entry Age** | **Last Follow-Up Age** | **Education** | **Smoking status** | **Comorbidity** |
| --- | --- | --- | --- | --- | --- | --- | --- |
| **ARIC** |  |  |  |  |  |  |  |
| Male White | survival >= 85 | 408 | 62.48(1.31) | 86.7(1.23) | 367(90.84) | 295(72.84) | 239(58.58) |
| Male Black | survival >= 85 | 63 | 62.97(1.34) | 86.94(1.36) | 34(53.97) | 47(74.6) | 31(49.21) |
| Female White | survival >= 85 | 506 | 62.42(1.34) | 86.78(1.27) | 459(91.07) | 216(42.77) | 221(43.68) |
| Female Black | survival >= 85 | 139 | 62.73(1.35) | 86.8(1.15) | 96(70.07) | 51(36.96) | 59(42.45) |
| All White | survival >= 85 | 914 | 62.45(1.32) | 86.75(1.26) | 826(90.97) | 511(56.15) | 460(50.33) |
| All Black | survival >= 85 | 202 | 62.81(1.35) | 86.84(1.22) | 130(65) | 98(48.76) | 90(44.55) |
| Male White | survival < 85 | 2091 | 57.14(5.25) | 71.77(7.51) | 1853(88.7) | 1761(84.22) | 1323(63.27) |
| Male Black | survival < 85 | 818 | 55.92(5.72) | 69.06(8.09) | 542(66.42) | 700(85.57) | 475(58.07) |
| Female White | survival < 85 | 1547 | 56.7(5.27) | 72.14(7.71) | 1431(92.5) | 1000(64.64) | 834(53.91) |
| Female Black | survival < 85 | 910 | 55.34(5.58) | 69.27(7.94) | 692(76.55) | 495(54.46) | 553(60.77) |
| All White | survival < 85 | 3638 | 56.95(5.26) | 71.93(7.6) | 3284(90.32) | 2761(75.89) | 2157(59.29) |
| All Black | survival < 85 | 1728 | 55.62(5.65) | 69.17(8.01) | 1234(71.74) | 1195(69.2) | 1028(59.49) |
| **CHS** |  |  |  |  |  |  |  |
| Male White | survival >= 85 | 718 | 78.54(5.05) | 89.09(3.29) | 591(82.54) | 438(61.09) | 474(66.02) |
| Male Black | survival >= 85 | 67 | 80.79(4.96) | 89.07(3.2) | 42(63.64) | 36(53.73) | 43(64.18) |
| Female White | survival >= 85 | 912 | 77.68(4.63) | 89.04(3.19) | 770(84.52) | 293(32.16) | 518(56.8) |
| Female Black | survival >= 85 | 154 | 79.86(4.32) | 89.8(3.51) | 92(60.13) | 44(28.57) | 93(60.39) |
| All White | survival >= 85 | 1630 | 78.06(4.84) | 89.06(3.23) | 1361(83.65) | 731(44.9) | 992(60.86) |
| All Black | survival >= 85 | 221 | 80.14(4.54) | 89.58(3.43) | 134(61.19) | 80(36.2) | 136(61.54) |
| Male White | survival < 85 | 819 | 72.12(4.18) | 78.67(4.25) | 678(82.78) | 616(75.4) | 651(79.49) |
| Male Black | survival < 85 | 127 | 71.74(4.36) | 77.17(4.57) | 96(76.19) | 100(81.97) | 95(74.8) |
| Female White | survival < 85 | 729 | 71.46(3.99) | 78.93(3.98) | 623(85.69) | 413(56.73) | 560(76.82) |
| Female Black | survival < 85 | 150 | 72.27(4.12) | 78.32(3.96) | 110(73.33) | 69(47.92) | 126(84) |
| All White | survival < 85 | 1548 | 71.81(4.11) | 78.79(4.12) | 1301(84.15) | 1029(66.6) | 1211(78.23) |
| All Black | survival < 85 | 277 | 72.03(4.23) | 77.79(4.28) | 206(74.64) | 169(63.53) | 221(79.78) |
| Male White | survival to the 90th percentile | 102 | 85.17(4.22) | 95.33(1.86) | 73(71.57) | 56(54.9) | 72(70.59) |
| Male Black | survival to the 90^th^ percentile | 15 | 87.2(3.95) | 93.96(2.17) | 7(50) | 10(66.67) | 6(40) |
| Female White | survival to the 90^th^ percentile | 47 | 86.83(4.3) | 97.41(2.25) | 33(70.21) | 5(10.64) | 26(55.32) |
| Female Black | survival to the 90^th^ percentile | 14 | 88.36(2.73) | 97.29(2.47) | 6(42.86) | 2(14.29) | 10(71.43) |
| All White | survival to the 90^th^ percentile | 149 | 85.69(4.3) | 95.99(2.21) | 106(71.14) | 61(40.94) | 98(65.77) |
| All Black | survival to the 90^th^ percentile | 29 | 87.76(3.41) | 95.57(2.84) | 13(46.43) | 12(41.38) | 16(55.17) |
| Male White | Didn’t survive to the 90^th^ percentile | 1164 | 74.43(5.45) | 81.5(5.76) | 952(81.79) | 833(71.75) | 910(78.18) |
| Male Black | Didn’t survive to the 90^th^ percentile | 157 | 73.45(5.45) | 79.2(5.91) | 112(71.79) | 113(74.34) | 119(75.8) |
| Female White | Didn’t survive to the 90^th^ percentile | 1146 | 74.46(5.64) | 82.54(5.96) | 965(84.43) | 545(47.64) | 859(74.96) |
| Female Black | Didn’t survive to the 90^th^ percentile | 195 | 74.3(5.45) | 80.76(5.78) | 135(69.59) | 78(41.27) | 166(85.13) |
| All White | Didn’t survive to the 90^th^ percentile | 2310 | 74.44(5.54) | 82.02(5.88) | 1917(83.09) | 1378(59.78) | 1769(76.58) |
| All Black | Didn’t survive to the 90^th^ percentile | 352 | 73.92(5.46) | 80.06(5.88) | 247(70.57) | 191(56.01) | 285(80.97) |

CHS: dbGaP version 7 of Cardiovascular Health Study

ARIC: dbGaP version 5 of Atherosclerosis Risk in Communities Study

Entry age/Last follow-up age: mean age (sd)

Comorbidity: presence of any of these major diseases (cancer, diabetes, CHD): N (percentage)

Education (high school or higher): N (percentage)

Smoking status (ever smoked): N (percentage)

**Supplementary Table 2 |** SNPs in the candidate genes available for this study after QC

| **GENE** | **Number of SNPs** | | **SNPs available in both ARIC and CHS CARe data** (genotyped on the IBC Chip, Illumina) |
| --- | --- | --- | --- |
|  | **CHS** | **ARIC** |  |
| **AKT1** | 3 | 3 | rs11849304 rs2494738 rs1130214 |
| **ATM** | 20 | 15 | rs4987874 rs4987876 rs4987886 rs3218693 rs4987923 rs11212570 rs1800056 rs664677 rs1800058 rs1800889 rs4988023 rs1801516 rs639923 rs374443 rs227041 |
| **PRKAA1** | 6 | 6 | rs249428 rs6882903 rs17239241 rs11747210 rs3805492 rs3805486 |
| **PRKAA2** | 8 | 7 | rs11206889 rs11579321 rs2796529 rs2746347 rs2746342 rs2796498 rs1418442 |
| **PRKAB2** | 5 | 4 | rs17159890 rs2304893 rs1036850 rs6689934 |
| **PRKAG1** | 2 | 2 | rs1126930 rs10875910 |
| **PRKAG2** | 127 | 102 | rs17714947 rs5016447 rs4726048 rs2302528 rs17715595 rs17173199 rs10215932 rs6946498 rs3789810 rs2538036 rs12535806 rs4726071 rs2079987 rs7796163 rs2727528 rs11970853 rs1860735 rs11982435 rs2727537 rs1860746 rs2024266 rs6970666 rs10275386 rs2536083 rs7778356 rs7780804 rs6952398 rs6967838 rs10235478 rs11978201 rs2374270 rs10480300 rs7805747 rs11980120 rs10224210 rs10224002 rs7792937 rs2536075 rs1860743 rs1104897 rs885273 rs7797862 rs11771414 rs17173258 rs6960717 rs11773668 rs6970522 rs7807769 rs7801616 rs2109782 rs868624 rs1104838 rs7800069 rs7784818 rs3934596 rs4725431 rs4725432 rs4418266 rs11773541 rs11981567 rs7782177 rs4726096 rs4128396 rs9648724 rs4442045 rs4726099 rs13224758 rs12703159 rs6969921 rs1881638 rs4582468 rs1881634 rs7455706 rs10952318 rs11768925 rs1881633 rs6975614 rs1881632 rs6962522 rs1881628 rs13310994 rs11770376 rs13225852 rs7805942 rs1108845 rs11773373 rs13238117 rs11764602 rs1881624 rs10277655 rs11772236 rs7795096 rs1881639 rs11762585 rs6961830 rs9640300 rs6961971 rs12703164 rs9640302 rs12703165 rs13240743 rs4725435 |
| **BAX** | 9 | 8 | rs1805419 rs4645886 rs4645887 rs1010104 rs2387583 rs4645900 rs905238 rs4645903 |
| **BCL2** | 140 | 97 | rs4987869 rs4987864 rs4987859 rs4987853 rs4987852 rs4987850 rs1564483 rs1016860 rs4987845 rs1982673 rs10503078 rs4941183 rs4987839 rs4987838 rs4987835 rs4987829 rs1531697 rs4987827 rs4456611 rs2046137 rs4987817 rs3943258 rs8095077 rs12457170 rs17756365 rs8099140 rs1026825 rs956572 rs7230970 rs899968 rs8097918 rs6567328 rs4987801 rs12454712 rs9962656 rs4987792 rs17679032 rs17070827 rs9959983 rs4987783 rs2062010 rs2062011 rs4987786 rs4987788 rs9955190 rs7243091 rs12957119 rs17841945 rs720321 rs12967026 rs954954 rs4987773 rs3826622 rs3810027 rs4987771 rs4987770 rs4987767 rs4987765 rs11152374 rs11663275 rs4987764 rs17685559 rs11872329 rs17070904 rs4987752 rs1944419 rs7226979 rs3744951 rs12457893 rs4987745 rs8085707 rs17070943 rs8094315 rs4987738 rs4987737 rs4987736 rs4987735 rs7242402 rs8089538 rs8089331 rs17070959 rs4987730 rs4987726 rs4987722 rs4987721 rs7232625 rs1381547 rs1381548 rs4987719 rs4941195 rs2850758 rs2849382 rs12458289 rs949037 rs2849380 rs1944422 rs1473418 |
| **CDK4** | 4 | 1 | rs2069507 |
| **CDK6** | 43 | 34 | rs4272 rs42031 rs42041 rs2237570 rs11771637 rs17688839 rs2282978 rs2282983 rs11765954 rs11773884 rs2106135 rs2282987 rs7781436 rs17164721 rs3731321 rs3731318 rs2237574 rs2282991 rs17690388 rs17164760 rs17164769 rs6952653 rs3731308 rs10 rs2237575 rs3731302 rs8 rs445 rs3731283 rs17147373 rs6951960 rs11974095 rs2158134 rs929244 |
| **CDKN1A** | 18 | 15 | rs3829963 rs733590 rs762623 rs762624 rs3176320 rs3176321 rs3176323 rs3176326 rs3176329 rs3176331 rs3176335 rs3176343 rs3176344 rs3176348 rs1059234 |
| **CDKN2A** | 15 | 14 | rs3731255 rs3088440 rs3731249 rs3731246 rs2811708 rs2811709 rs4074785 rs3731222 rs3731218 rs3731217 rs3731201 rs3731198 rs7036656 rs3731194 |
| **CDKN2B** | 6 | 3 | rs3217986 rs2069422 rs3217973 |
| **FAS** | 41 | 40 | rs4934434 rs3758483 rs9658674 rs1800682 rs9658676 rs10509561 rs7097467 rs2031610 rs7909414 rs9658683 rs4345878 rs9658699 rs9658702 rs7094676 rs9658705 rs9658706 rs1571011 rs1571012 rs9658713 rs2147420 rs1159120 rs2147418 rs3218619 rs9658734 rs9658735 rs9658741 rs9658742 rs9658750 rs2031611 rs7910435 rs1926190 rs9658761 rs1571020 rs1571019 rs9658769 rs9658771 rs1051070 rs1468063 rs9658791 rs9658792 |
| **FOXO1** | 11 | 10 | rs2701858 rs17446614 rs2984121 rs3858869 rs4943795 rs3908774 rs12876443 rs7981045 rs7139990 rs9603776 |
| **FOXO3** | 11 | 8 | rs9372190 rs9384681 rs2883881 rs13220810 rs2764262 rs1935957 rs12203834 rs3800230 |
| **GHR** | 30 | 23 | rs17574527 rs17574650 rs17636762 rs2940923 rs6451620 rs11744988 rs2972393 rs6883523 rs6898743 rs12109702 rs7735231 rs7727694 rs4594864 rs6886047 rs7731163 rs4994772 rs6878461 rs6451637 rs6894403 rs7712778 rs10805665 rs2973015 rs6180 |
| **HIF1A** | 4 | 4 | rs2181600 rs1957755 rs17099141 rs11549465 |
| **IGF1** | 35 | 35 | rs6219 rs3730192 rs6214 rs3730204 rs1520220 rs5742696 rs5742694 rs5742692 rs11111265 rs9308315 rs17882373 rs5009837 rs11111267 rs7300373 rs5742658 rs5742657 rs7136446 rs10735380 rs12310428 rs12299353 rs11831436 rs7971494 rs2195240 rs5742627 rs7956547 rs17884900 rs1019731 rs5742624 rs5742620 rs5742615 rs17032634 rs17882264 rs11831702 rs5742614 rs5742612 |
| **IGF1R** | 203 | 168 | rs874305 rs4966010 rs13329348 rs13329408 rs11247361 rs10902605 rs10902606 rs11854132 rs11247367 rs1976667 rs7170290 rs11630647 rs10438474 rs4966013 rs7166348 rs4966014 rs4966015 rs8037116 rs7174918 rs12901570 rs3803477 rs3803476 rs3759906 rs28401700 rs8038015 rs11857366 rs1543106 rs4966017 rs932071 rs4616271 rs875686 rs4966018 rs11633294 rs4393553 rs1567810 rs3743264 rs8037467 rs7176092 rs11855223 rs11632952 rs6598542 rs12898502 rs8041224 rs2175796 rs8038291 rs2048639 rs8037855 rs7175387 rs11247370 rs925585 rs11855167 rs11857362 rs884663 rs8024123 rs1513643 rs2175799 rs4267291 rs4966028 rs907806 rs3759908 rs6598554 rs12443294 rs12898932 rs2684761 rs2670501 rs2684763 rs7174707 rs7183648 rs2715436 rs2684777 rs1879612 rs8030950 rs7164105 rs12594847 rs2684779 rs2684780 rs11247377 rs2715442 rs11247378 rs28672978 rs4966035 rs4966036 rs11247379 rs11630259 rs6650547 rs1357112 rs1879613 rs7170203 rs11247380 rs3743256 rs3743258 rs3743259 rs4966038 rs867431 rs1980269 rs12439656 rs2715417 rs2684811 rs2139924 rs2228531 rs951715 rs7169544 rs3784604 rs3784605 rs3784606 rs2684808 rs28612945 rs10048024 rs2715425 rs9920651 rs7182342 rs4966042 rs2684807 rs28637929 rs2684806 rs3743262 rs12442093 rs2684805 rs34688626 rs4966044 rs8039599 rs2715429 rs2715428 rs2684803 rs2684802 rs1546713 rs4486868 rs4966046 rs7162336 rs12438495 rs2229765 rs4987179 rs28401726 rs2293117 rs1521479 rs2684799 rs28631431 rs8030050 rs1568501 rs2715419 rs2684794 rs1568502 rs12916884 rs10438491 rs7166565 rs2684791 rs4966048 rs2684789 rs17847195 rs2715439 rs939626 rs7167580 rs8034284 rs12148482 rs9672254 rs12591122 rs9672965 rs8038415 rs8025801 rs12440962 rs3743253 rs2593053 rs34804698 rs702497 rs2016873 rs1058696 rs2684788 rs2654981 |
| **KL** | 24 | 18 | rs562020 rs495392 rs385564 rs9526961 rs563925 rs577912 rs9526990 rs472875 rs2320762 rs9536282 rs1888057 rs684868 rs7986435 rs9536314 rs9527025 rs2149860 rs9596717 rs650439 |
| **MYC** | 5 | 3 | rs4645946 rs4645959 rs10110283 |
| **NFKB1** | 60 | 56 | rs3774933 rs4647965 rs4647968 rs4647972 rs1598857 rs1599961 rs1585213 rs1598856 rs4647987 rs230532 rs230528 rs230525 rs4648006 rs4648008 rs17032779 rs230515 rs4648011 rs13117745 rs230506 rs230491 rs17032815 rs4648022 rs230541 rs4648026 rs4648037 rs4648038 rs4648039 rs4648047 rs1287 rs1609993 rs4648049 rs4648058 rs4648064 rs4648069 rs4648072 rs4648073 rs3774963 rs4648075 rs3774964 rs3774965 rs12509403 rs4648090 rs4648093 rs4648095 rs3774968 rs4648104 rs4648110 rs3817685 rs4648123 rs4648126 rs4648127 rs4648128 rs230547 rs4648133 rs4648135 rs1609798 |
| **NFKB2** | 5 | 4 | rs12772374 rs7897947 rs11574851 rs7077329 |
| **PIK3CA** | 11 | 9 | rs7621329 rs9871768 rs2699905 rs7628855 rs7651265 rs7640662 rs13082485 rs17592582 rs2230461 |
| **PIK3CB** | 14 | 7 | rs361088 rs2305268 rs2595932 rs693293 rs385254 rs10513055 rs500687 |
| **PPARGC1A** | 47 | 41 | rs6821591 rs12650562 rs7682765 rs2932965 rs1873532 rs3736265 rs2932971 rs2932976 rs2932977 rs3774909 rs2290604 rs16874209 rs4619879 rs6838835 rs12374310 rs12374408 rs7672915 rs11941854 rs12645360 rs12640088 rs4697426 rs7656250 rs7677000 rs16874260 rs6838600 rs4361373 rs13128633 rs17637318 rs11734408 rs2305682 rs12500214 rs2946385 rs2970873 rs2970872 rs3774902 rs16874290 rs7695542 rs2970870 rs7657071 rs7666134 rs17582609 |
| **PTEN** | 8 | 7 | rs1234220 rs17106896 rs12357281 rs2735343 rs9783238 rs926091 rs11202607 |
| **RPS6KB1** | 5 | 4 | rs8071475 rs16943954 rs180520 rs180515 |
| **SIRT1** | 13 | 12 | rs35706870 rs3740051 rs33957861 rs34234131 rs35409355 rs34071962 rs2273773 rs3818292 rs34414573 rs34347509 rs10997874 rs2234975 |
| **TP53** | 5 | 3 | rs12951053 rs2909430 rs8079544 |
| **TSC2** | 2 | 2 | rs8063461 rs30259 |
| **TGFB1** | 8 | 8 | rs8179181 rs8110090 rs11466334 rs1800472 rs4803455 rs2241716 rs2241715 rs1800469 |
| **TGFBR2** | 105 | 90 | rs3087463 rs11466480 rs1835538 rs1991657 rs9310938 rs17029848 rs9790292 rs17025760 rs1036095 rs9844092 rs6773330 rs17025785 rs4522809 rs4955212 rs17025788 rs6809777 rs17025795 rs12490899 rs17025824 rs12491780 rs11466491 rs1947687 rs1431131 rs2043139 rs11924422 rs9858487 rs17025857 rs9867701 rs17838698 rs17025862 rs1155705 rs11466493 rs13081020 rs891595 rs1808602 rs2005061 rs1078985 rs6790706 rs11466500 rs17025960 rs9831477 rs1036097 rs3773626 rs1019856 rs3773632 rs3773634 rs17026037 rs2082225 rs9838479 rs9838771 rs995435 rs6550007 rs9310939 rs6792117 rs9842186 rs3773644 rs11466511 rs2229102 rs2228048 rs11466517 rs3773646 rs1123686 rs11466521 rs3773648 rs3773649 rs17026203 rs2116142 rs3773650 rs3773651 rs2043136 rs2372212 rs4955189 rs9310940 rs3773656 rs1346907 rs17026240 rs876687 rs876688 rs3773660 rs3773661 rs9838539 rs11466522 rs9843942 rs11466525 rs3773662 rs3773663 rs304839 rs2276767 rs1803446 rs11466536 |
| **Total** | **1053** | **863** |  |

**Supplementary Table 3|** Main protein/metabolite classes shown in Figure 2 in the text

| 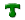   \| ligands \| \| --- \| | 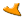  proteases | \| receptors 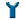 \| \| --- \| |
| --- | --- | --- | --- | --- |
| \| kinases 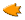 \| \| --- \| | 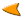  enzymes | 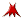   \| transcription factors \| \| --- \| |
| \| 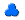 \| \| --- \|   other generic proteins |   binding proteins | \| 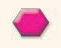  compounds/  metabolites \| \| --- \| |
